# Psychological Impact of COVID-19 on Children and Adolescents: A Narrative Review of Mental Health Challenges, Interventions, and Long-Term Trajectories

**DOI:** 10.7759/cureus.81840

**Published:** 2025-04-07

**Authors:** Purushottam Lal, Surendra Gupta

**Affiliations:** 1 Pediatrics, Mohawk Valley Health System, Utica, USA; 2 Pediatrics, University of New England College of Osteopathic Medicine, Biddeford, USA; 3 Pediatrics, Clinica Sierra Vista, Fresno, USA

**Keywords:** anxiety, cognitive behavioral management, covid-19, depression, mental health

## Abstract

The COVID-19 pandemic has significantly impacted the mental health and well-being of children and adolescents globally. This comprehensive narrative review synthesizes current research on the pandemic's psychological effects on young people, examining emotional distress, behavioral changes, sleep disturbances, educational disruptions, and the exacerbation of pre-existing mental health conditions. A systematic literature search was conducted using PubMed, MEDLINE, PsycINFO, CINAHL, and Web of Science, covering articles published from January 2020 to December 2024. Studies focusing on children and adolescents (aged 0-18 years) that examined mental health outcomes related to the COVID-19 pandemic were included. Data extraction was performed using a standardized form, and a narrative synthesis approach was employed to analyze and integrate the findings. Key findings reveal a substantial increase in anxiety, depression, and post-traumatic stress disorder among children and adolescents during the pandemic. School closures, social isolation, and disrupted routines have contributed to these mental health challenges. Sleep patterns have been notably affected, with delayed bedtimes, increased sleep disturbances, and a higher prevalence of insomnia and nightmares. The pandemic has widened educational disparities, particularly affecting children from disadvantaged backgrounds. Individuals with pre-existing mental health conditions have experienced exacerbated symptoms and faced challenges in accessing care. Various interventions, including cognitive behavioral therapy, social-emotional learning programs, and digital mental health support, have shown promise in mitigating the pandemic's psychological impact on young people. Children were more affected, as they faced a global crisis for the first time with limited coping skills. Disruptions in routine, social isolation, and family stressors heightened anxiety, depression, and behavioral issues, making them more affected. This review emphasizes the need for continued research, targeted interventions, and policy development to address the long-term mental health consequences of the COVID-19 pandemic on children and adolescents.

## Introduction and background

The COVID-19 pandemic, triggered by the novel SARS-CoV-2, has profoundly impacted global health and societal well-being. Beyond the immediate physical health crisis, the pandemic has significantly contributed to a surge in mental health challenges. Children and adolescents, who are in critical developmental stages, have been disproportionately affected by the psychosocial consequences of this unprecedented global event [1].

The pandemic's impact on young people's mental health is multifaceted, encompassing disruptions to education, social isolation, increased family stress, and heightened exposure to trauma and loss [2,3]. School closures implemented in 193 countries affected more than 1.59 billion students, disrupting education and social development [2]. The transition to remote learning presented additional challenges, including unequal access to technology, decreased engagement, and a lack of personalized support [4]. Social isolation, coupled with the cancellation of extracurricular activities and social interactions, further deprived young people of opportunities for crucial social connection [5]. These challenges have exacerbated existing inequalities, disproportionately affecting vulnerable children and adolescents who already face significant hardships [6].

Post-traumatic stress disorder (PTSD) symptoms in children and adolescents during the COVID-19 pandemic were assessed using validated tools. Marques de Miranda et al. identified three studies that evaluated post-traumatic stress symptoms using standardized scales, including the Impact of Event Scale-Revised and the PTSD Checklist-Civilian Version. Additionally, broader mental health assessments, such as the 12-item General Health Questionnaire (GHQ-12) and the COVID-19 Peritraumatic Distress Index (CPDI), were used to measure psychological distress, including anxiety, depression, and cognitive changes. Assessing PTSD symptoms during an ongoing crisis is challenging due to overlapping anxiety and distress, but these tools provide structured methods to evaluate trauma-related responses [7]. Disruption of routines, loss of social connections, and heightened familial stress have all contributed to this surge in mental health issues [8]. Economic instability within families, due to job losses or financial constraints, has further compounded stress levels in children, increasing their vulnerability to mental health challenges. Increased screen time during remote learning and digital interactions has been linked to behavioral issues, sleep disturbances, and feelings of isolation [7]. The fear of illness, loss of loved ones, and witnessing grief within communities have further heightened trauma levels among young individuals. Reduced access to extracurricular activities, which often serve as emotional outlets, has also left many children and adolescents with limited coping mechanisms, worsening their psychological struggles [8].

This review synthesizes the existing literature on the impact of the COVID-19 pandemic on child and adolescent mental health. It examines the effects of school closures, social isolation, family stress, and trauma exposure across different regions, including North America, Europe, Asia, and South America [6,7,4,9]. Specific mental health outcomes include anxiety, depression, PTSD, and other challenges related to the pandemic [1,7]. The review also highlights interventions and strategies to support young people during and after the pandemic, emphasizing the roles of families, schools, and communities in promoting mental well-being [5,8]. By integrating available evidence, this review aims to inform policy and practice to mitigate the pandemic's short- and long-term mental health consequences for children and adolescents worldwide.

## Review

Materials and methods

A systematic literature search was conducted using PubMed, MEDLINE, PsycINFO, CINAHL, and Web of Science, covering articles published from January 2020 to December 2024. The search strategy included key terms such as "COVID-19", "SARS-CoV-2", "children", "adolescents", "mental health", "psychological impact", "anxiety", "depression", "stress", "coping strategies", "interventions", and "long-term outcomes", with appropriate Boolean operators (e.g., AND, OR) to refine the search results.

This narrative review included peer-reviewed original research articles, systematic reviews, meta-analyses, and longitudinal studies focusing on the psychological impact of COVID-19 on children and adolescents. Quantitative and qualitative studies examining mental health challenges, coping mechanisms, and interventions were considered. Studies were excluded if they focused solely on adult populations, did not directly address mental health outcomes, were non-empirical articles, or lacked methodological rigor.

Data extraction and analysis involved two independent reviewers screening titles and abstracts, with full-text assessment of potentially eligible studies. A standardized form was used to capture information on study characteristics, sample size, and demographics, including children and adolescents less than 18 years of age, methodology, mental health outcomes, key findings, and limitations. A narrative synthesis approach was employed to analyze and integrate the findings, allowing for a comprehensive examination of diverse outcomes and methodologies.

Quality assessment

The studies were critically appraised for internal validity, consistency in outcome reporting, and alignment with best practices in pediatric obesity research. Ethical considerations were addressed by ensuring all included studies had appropriate ethical approvals and adhered to guidelines for research involving children and adolescents.

The findings were organized thematically, addressing key areas of impact such as emotional distress, behavioral changes, sleep disturbances, educational disruptions, and the impact on pre-existing mental health conditions. This comprehensive methodology provided a rigorous examination of the current literature on the mental health impact of the COVID-19 pandemic on children and adolescents, offering a solid foundation for understanding the scope and nature of these effects.

Clinical aspects

Impact on Mental Health

The COVID-19 pandemic has significantly impacted children's mental health, leading to increased levels of fear, anxiety, and grief. A systematic review analyzing literature from December 2019 to November 2020 found substantial emotional distress among children, with symptoms such as anxiety and irritability [10]. Additionally, a narrative review highlighted the rise in child maltreatment during the pandemic, linking it to parental job loss and increased caregiver stress [11]. The mental health challenges associated with school closures were also emphasized, with findings indicating heightened anxiety and depression among children due to disrupted routines and increased screen time [12]. A cross-sectional survey conducted in Saudi Arabia revealed that 71.5% of children exhibited PTSD symptoms during the pandemic, underscoring the critical need for mental health support [13].

Furthermore, an article on loss and resilience proposed that families adapt to the trauma of loss through shared belief systems, facilitating meaning-making during crises [14]. Finally, a meta-analysis synthesizing data from over 80,000 youth globally revealed that clinically elevated symptoms of depression and anxiety were significantly higher during the pandemic than before it [15]. Collectively, these studies illustrate the profound psychological effects of the COVID-19 pandemic on children's mental health, emphasizing increased rates of anxiety, depression, PTSD, and maltreatment associated with various stressors linked to the pandemic environment.

Emotional Distress Among Children and Adolescents During the COVID-19 Pandemic

The COVID-19 pandemic had a profound emotional impact on children and adolescents, even among those who did not meet the criteria for a clinical diagnosis. The uncertainty, fear, and disruption associated with the pandemic led to increased anxiety, worry, sadness, and irritability in many young people [16-18]. Children often expressed their distress through behavioral changes, including clinginess, altered play patterns, and sleep disturbances [19,20].

Quantitative studies provide evidence of the significant emotional toll of the pandemic. A meta-analysis of 29 studies involving 80,879 youth globally reported pooled prevalence estimates of clinically elevated depression and anxiety in children and adolescents at 25.2% and 20.5%, respectively, which is approximately double the pre-pandemic rates [21]. In China, 22.6% of children isolated at home exhibited symptoms of depression, while in Germany, 30.1% of adolescents aged 11 to 17 reported symptoms of generalized anxiety, compared to 14.9% before the outbreak [22,23].

A large-scale cross-sectional study in China involving 1,199,320 children and adolescents found a 10.5% prevalence of self-reported psychological distress during the pandemic, using the GHQ-12 to assess distress [24]. Emotional responses varied by age group: younger children displayed increased clinginess and fear, school-aged children exhibited heightened anxiety and depression, and adolescents experienced elevated levels of anxiety, depression, and impulsivity [25,26]. These findings highlight the widespread emotional toll of the pandemic on children and adolescents, emphasizing the need for targeted mental health interventions and support systems for this vulnerable population [27,28].

Behavioral Changes in Children During the COVID-19 Pandemic

The COVID-19 pandemic and associated lockdowns significantly impacted children's behavior. Reduced physical activity (PA) and increased sedentary behaviors (SBs), particularly screen time, were notable. Xiang et al. found a drastic decline in PA levels among children in Shanghai, China, with physical inactivity rising from 21.3% to 65.6% during the pandemic [29]. Similarly, screen time increased substantially, with an average rise of 30 hours per week, especially for recreational activities [29]. This increase in SB was echoed by Bekelman et al. who observed a significant rise in daily screen time among children across various racial and ethnic groups, highlighting disparities in health behaviors during the pandemic [30].

Behavioral problems, including anxiety, depression, irritability, and inattention, surged as shown in table 1. A systematic review by Panda et al. estimated that approximately 42.3% of children experienced irritability, while 34.5% and 41.7% faced anxiety and depression, respectively [31]. These psychological challenges were compounded by prolonged confinement and disrupted routines, as highlighted by Liu et al., where 4.7-10.3% of children exhibited behavioral issues such as hyperactivity and emotional distress during home quarantine [32].

**Table 1:** Summary of studies on COVID-19's impact on children's and adolescents' mental health and sleep CI, confidence interval; HRQoL, health-related quality of life

Study	Study Focus	Population (Age in Years)	Key Findings	Limitations	Study Design	Region
Racine et al. (2021) [15]	Depression and anxiety	29 studies, 80,879 participants (<18 years)	Depression: 25.2% (95% CI: 21.2%-29.7%); anxiety: 20.5% (95% CI: 17.2%-24.4%); higher prevalence with later data collection, female gender, and older age	Limited to English studies and no long-term impact assessment	Meta-analysis	International
Ravens-Sieberer et al. (2021) [23]	Mental health impact	Representative cohort of children and adolescents (7-17 years)	Low HRQoL: 40.2% (vs. 15.3% pre-pandemic), increased mental health issues: 17.8% (vs. 9.9%), disadvantaged backgrounds more affected	Limited generalizability due to unique context of Germany	Longitudinal	Germany
Xie et al. (2020) [24]	Mental health	1,784 students, grades 2-6	Depression: 22.6%, anxiety: 18.9%, higher depression risk in Wuhan than Huangshi, optimism about COVID-19 reduced depression risk	Self-reported data, lack of baseline information	Cross-sectional	China
Panda et al. (2021) [31]	Psychological impact	15 studies, 22,996 children (<18 years)	Anxiety: 34.5%; depression: 41.7%, irritability: 42.3%, fear of COVID-19: 22.5%, sleep disturbances: 21.3%	Heterogeneity in measurement tools and populations	Meta-analysis	International
Liu et al. (2020) [32]	Behavioral issues	1,264 children, grades 2-6 (7-12 years)	Behavioral problems: 4.7% to 10.3%, physical activity linked to fewer behavior problems	Self-reported data and lack of pre-pandemic baseline	Cross-sectional	China
Bruni et al. (2021) [33]	Sleep changes	4,314 subjects (1–18 years)	Delayed bedtime and wake time, increased sleep disturbances in younger children, increased screen time during lockdown	Lack of pre-pandemic sleep data, self-reported issues	Cross-sectional	Italy
Bothe et al. (2022) [34]	Sleep and lifestyle changes	2,290 children and adolescents (6–18 years)	Delayed sleep and wake times; increased screen time and decreased physical activity and daylight exposure; increased insomnia, nightmares, and daytime sleepiness; sleeping problems correlated with COVID-19 anxiety	Cross-sectional	Austria	
Sharma et al. (2021) [35]	Sleep disturbances	16 studies, various age groups (<18 years)	Sleep disturbances prevalence: 54% (95% CI: 50-57%), not meeting sleep recommendations: 49%	Heterogeneous outcome measures and reliance on online surveys	Meta-analysis	International

Children with pre-existing behavioral disorders were particularly vulnerable. Colizzi et al. observed worsening symptoms in children with autism spectrum disorder (ASD), while Orgilés et al. documented increased stress among youth in Italy and Spain [36,37]. Despite these challenges, some positive changes were reported. Bekelman et al. noted improved sleep durations among children who previously lacked sufficient rest [30]. These behavioral shifts underscore the need for targeted interventions to promote PA and address psychological challenges during crises [38]. Restoring healthy routines and providing support for children and their families are essential to mitigate long-term adverse effects.

Impact on Children's Sleep Patterns and Disturbances

The COVID-19 pandemic has significantly impacted children's sleep patterns and behaviors. Multiple studies have reported changes in sleep schedules, with delayed bedtimes and wake times observed across different age groups [33-35]. While some studies found increased sleep duration [33], others reported no significant changes [34]. Sleep disturbances, including difficulty falling asleep, anxiety at bedtime, night awakenings, and nightmares, increased in prevalence, particularly among younger children [35,39]. The pooled prevalence of any sleep disturbance during the pandemic was estimated at 54% [39].

Children of healthcare workers appeared to be more affected, with greater impairment in sleep habits compared to children of non-healthcare workers [40]. Factors such as increased screen time, decreased PA, and reduced daylight exposure were associated with sleep changes [33,38]. Additionally, COVID-19-related anxiety correlated with sleep problems and reduced quality of life [41].

Educational Disruptions and Challenges During the COVID-19 Pandemic

The COVID-19 pandemic has exacerbated existing inequalities and created new challenges for students, particularly those from marginalized backgrounds [42,43]. Nearly 15% of children in the United States of America (USA) lack reliable internet access, hindering participation in online learning for low-income families [42]. Key challenges include learning losses, decreased student engagement, and difficulties in assessing progress, especially for conditions such as attention deficit hyperactivity disorder (ADHD) and ASD [42,43].

The pandemic has also increased anxiety and stress among students, adversely affecting academic performance [43]. Cachón-Zagalaz et al. highlighted concerns about the impact of prolonged confinement on children's physical development [44]. Many parents, particularly essential workers, have struggled to support home learning [42]. While some high-achieving students maintained performance levels, many disadvantaged students fell behind [43].

To address these challenges, researchers have recommended targeted support for vulnerable students, investment in technology and teacher training, and the development of safe reopening strategies [42,43]. Esposito et al. emphasized the importance of comprehensive safety measures during school reopening [45]. Putra et al. highlighted the need for enhanced support for students and families adapting to the "new normal" in education [46]. Further research is necessary to inform interventions addressing the long-term effects of the pandemic on children's education and development [45,46].

Impact of the COVID-19 Pandemic on Individuals With Pre-existing Mental Health Conditions

The COVID-19 pandemic and its associated restrictions have profoundly impacted individuals with pre-existing mental health conditions. Multiple studies reveal a nuanced picture of heightened challenges alongside resilience. Research by Murphy et al. identified significant effects, including increased social isolation, loneliness, reduced access to health services, and exacerbation of symptoms [47]. Individuals with conditions such as depression, anxiety, and schizophrenia frequently experienced a worsening of their symptoms. There was also a rise in self-harming behaviors and suicidality [47]. A systematic review by Carvalho et al. involving 49 studies concluded that people with pre-existing psychiatric conditions faced more psychological distress and anxiety during the pandemic than those without such diagnoses [48]. Specific groups, including individuals with eating disorders and obsessive-compulsive disorder, faced exacerbated symptoms [49,50].

Children and adolescents with pre-existing mental or somatic conditions were particularly affected. Zijlmans et al. studied psychiatric participants aged 8-18 years receiving care for conditions such as autism, depression, and ADHD at four tertiary academic child and adolescent psychiatric centers in the Netherlands. The pediatric participants included children with chronic somatic conditions such as juvenile idiopathic arthritis, endocrinological diseases, and cystic fibrosis, who were under treatment at the Emma Children's Hospital Amsterdam UMC during the first lockdown in the Netherlands. They found significant issues among the psychiatric group, while the pediatric group reported comparatively fewer problems [51]. Similarly, Palacio-Ortíz et al. reviewed the pandemic’s impact on children and adolescents with pre-existing psychiatric disorders, noting increased symptoms in conditions such as ADHD, ASD and anxiety [52].

Lewis et al. investigated the experiences of 2,869 individuals with pre-existing mental illnesses in the United Kingdom, finding that 60% of participants reported worsened mental health during the pandemic [53]. Studies by Pierce et al., Godara et al., and Santos et al. consistently highlighted stress as a trigger for psychiatric disorders, including anxiety, mood, and psychotic disorders [54-56]. Additionally, the Household Pulse Survey in the USA reported an increase in anxiety and depression symptoms, with pre-existing psychiatric conditions, female sex, and concerns about infection being significant risk factors [57].

Treatment Approaches

As the pandemic evolved, early studies identified stress, anxiety, and depression as prevalent issues among vulnerable groups, such as healthcare workers, highlighting risk and protective factors. Continued interventions, including telehealth and self-care practices, are essential to support the mental health of individuals with pre-existing conditions [58].

The COVID-19 pandemic has significantly impacted children's mental health, prompting the development of various interventions to address these challenges. These interventions aim to support emotional well-being, provide coping strategies, and mitigate the pandemic's negative psychological effects on children and adolescents. Ranging from school-based programs to technology-assisted support and family-focused initiatives, these approaches seek to provide comprehensive mental health care tailored to the unique circumstances created by the pandemic.

Cognitive Behavioral Management

Cognitive behavioral therapy (CBT) has emerged as an effective, evidence-based approach for managing anxiety, depression, and stress, especially during the COVID-19 pandemic. CBT enhances self-awareness by helping individuals recognize and challenge distorted thinking patterns using constructive cognitive models, ultimately fostering resilience and improved coping mechanisms [58,59]. The therapy has shown significant benefits in improving sleep, reducing fatigue, and enhancing overall quality of life, particularly when adapted for remote delivery via telehealth platforms, which ensures accessibility and continuity of care during social distancing. Studies indicate that remotely delivered CBT yields outcomes comparable to in-person therapy [60,61]. Smartphone-based CBT applications have proven to be particularly effective, offering reminders, virtual peer support, and enhanced self-adherence while catering to individual needs [62,63].

Despite potential barriers such as lifestyle adjustments and adherence challenges, even modest engagement in CBT interventions can result in substantial improvements in emotional and psychological well-being [64,65]. This flexibility and adaptability make CBT a valuable resource in mitigating the psychological impact of crises like the COVID-19 pandemic [66,67].

Educational and Social Management

Social-emotional learning (SEL) programs have proven to be highly effective in supporting children's mental health during the COVID-19 pandemic. These programs provide preventive mental and emotional health education through evidence-based curricula that focus on key competencies such as self-awareness, self-management, social awareness, relationship skills, and responsible decision-making (Simmon, Social-Emotional Learning and Teachers’ Perceptions Using the Collaborative for Academic, Social, and Emotional Learning Framework, 2024). A systematic review and meta-analysis indicated that children who participated in SEL curricula showed significant improvements in social and emotional competence, behavioral self-regulation, and early learning skills, along with reductions in behavioral and emotional difficulties compared to control groups [68]. The emphasis on emotional competence, which is defined as the ability to identify and manage one’s own emotions and understand the emotions of others, has been particularly beneficial during this time, as it is associated with better school performance and resilience against stressors [69,70]. Although SEL programs have demonstrated clear benefits, there remains a gap in their adaptation for refugee children. Recent initiatives have begun to address this by using frameworks such as intervention mapping to develop culturally specific SEL programs that incorporate trauma-informed wellness components tailored for refugee children during the pandemic [71,72]. These adaptations not only meet the unique needs of these vulnerable populations but also foster community engagement and trust in mental health initiatives.

Technology-Assisted Management

During the COVID-19 pandemic, various digital technologies played a crucial role in supporting children's mental health as traditional avenues for social interaction and mental health services were severely restricted. Telehealth emerged as a vital resource, allowing children to access mental health care remotely, which proved particularly beneficial for those experiencing anxiety and depression due to isolation and uncertainty [73,74]. Additionally, mobile applications for mental health support provided tools for self-management and coping strategies, enabling children to engage in activities that fostered emotional well-being [75]. Virtual reality and serious games were also used to create immersive experiences that helped children process their emotions and connect with peers in a safe environment [76]. Furthermore, online platforms facilitated continued education and social interaction, which were essential in mitigating feelings of loneliness and disconnection during lockdowns [77]. However, it is important to note that while these technologies provided significant benefits, they also contributed to increased screen time and potential negative effects, including digital fatigue, social disconnection, and increased cyberbullying risks, which can affect mental health, highlighting the need for balanced usage [78,79]. 

## Conclusions

The COVID-19 pandemic has significantly impacted the mental health of children and adolescents, contributing to social-emotional delays, academic challenges, and increased vulnerability to psychological distress. While these effects may persist, targeted interventions can help mitigate long-term consequences. This review synthesizes findings from diverse studies, offering a comprehensive overview of the pandemic’s psychological effects. However, this review specifically focuses on the psychological impact of the pandemic, analyzing the effects of lockdowns, social isolation, school closures, and disrupted routines. While the neurological implications of SARS-CoV-2 have been explored in other research contexts, these aspects fall outside the scope of our review. Future research should explore the interplay between the virus’s direct neurological effects and its psychological consequences for a more comprehensive understanding. Additional limitations include variability in study methodologies, reliance on self-reported assessments, and a lack of long-term follow-up data.

To support children’s mental well-being, future strategies should include accessible mental health support systems, school-based counseling, and SEL integration. Maintaining structure, promoting hybrid learning models, and ensuring equitable access to mental health services for vulnerable populations are also crucial. Regular screenings, interdisciplinary collaboration, and digital literacy promotion will further aid in crisis preparedness. Longitudinal research is essential to guide evidence-based policies, ensuring that future generations can recover, adapt, and build resilience in the face of global crises.
